# Impact of heavy pruning on development and photosynthesis of *Tilia cordata* Mill. trees

**DOI:** 10.1371/journal.pone.0256465

**Published:** 2021-08-23

**Authors:** Marzena Suchocka, Tatiana Swoczyna, Joanna Kosno-Jończy, Hazem M. Kalaji

**Affiliations:** 1 Department of Landscape Architecture, Institute of Environmental Engineering, Warsaw University of Life Sciences–SGGW, Warsaw, Poland; 2 Department of Environment Protection and Dendrology, Institute of Horticultural Sciences, Warsaw University of Life Sciences–SGGW, Warszawa, Poland; 3 Department of Plant Physiology, Institute of Biology, Warsaw University of Life Sciences–SGGW, Warszawa, Poland; University of Hyderabad School of Life Sciences, INDIA

## Abstract

Tree pruning is carried out to reduce conflict with infrastructure, buildings, and any other human activity. However, heavy pruning may result in a diminished tree crown capacity for sugar production and exposure to fungal infection. This risk leads to a decrease in tree stability or vigour. In this work, we analysed the effect of heavy pruning of roadside trees on the photosynthetic performance process compared to neighbouring unpruned trees. Four years of tree crown growth was studied by terrestrial imaging. Tree vitality (Roloff’s classification) and risk (Visual Tree Assessment) were evaluated. Over-pruned trees showed intensified photosynthetic efficiency during the growing season following pruning. Particularly ET_0_/TR_0_ and PI_ABS_ tended to increase in pruned trees while higher F_v_/F_m_ was noted only in late October, suggesting delayed leaf senescence. After four years, pruned trees rebuilt their crowns, however not in their entirety. Results obtained from biometric, vitality, and risk assessment showed high differentiation in pruned tree crown recovery. Our results revealed that despite the intensified efforts of trees to recover from wounding effects, severe pruning evokes dieback occurrence and a higher risk of failure in mature trees.

## Introduction

Roadside trees are an essential part of the rural landscape. Tree-lined country roads fulfil some technical functions; trees drain and stabilise the road and the verges, shelter travellers from wind and provide shade in unsheltered sunny areas [1]. Moreover, they offer a valuable windbreak in the open countryside, helping to prevent soil erosion caused by the wind. They are a vital habitat for wildlife, act as shelters for wild animals, provide ecological corridors and support endangered species conservation [2–6]. Trees along roads in the rural landscape add aesthetic value, act as scenic routes, and create a landscape identity both for site residents and tourists [1,7].

The small-leaved lime (*Tilia cordata* Mill.) is a popular tree species for landscaping and urban environments [8–10]. Moreover, due to its wide ecological tolerance, this species could become vital in forestry and urban forestry, in the face of climate change [11]. Over fifty years, researchers have demonstrated that there has been a loss of trees in the urban environment. An example of this [12], can be seen in the five species with the highest loss in Warsaw. Three *Tilia* species have become endangered, among them *Tilia cordata*, which is probably a result of challenging site conditions, but improper pruning could potentially be an important factor causing the decline of lime trees.

Critical crown loss is defined as the loss of more than 50% of the branches [13,14]. Loss of such a large part of the crown leads to a weakening of tree vitality or is devastating to the tree’s structural integrity, posing a risk of failure. Proper tree pruning reduces conflicts with infrastructure and buildings, wind resistance and the risk of branches falling on objects and people [15]. Improper pruning leads to the poor appearance of the trees, leaves a large wound that will need a long time to recover, and exposes the tree to infections (parasitic fungi) that may decrease the tree’s stability (e.g. *Fomes fomentarius*) or vigour (e.g. *Armillaria* ssp.). Fungal decay can weaken the physical strength of the wood and may pose a danger to the public or property [16–18]. The fear of trees falling is the primary motivation for topping. Campanella [19], Fazio and Krumpe [20], Close et al. [21] and Zakaria [22] show that private homeowners or professional employees choose poor pruning practices such as topping because of insufficient experience, knowledge, and awareness of the negative impact of improper cuts on trees. Numerous investigators recommend avoiding radical pruning [20,23–29]. Others claim that only by raising the level of awareness in terms of knowledge, skills as well as attitudes could potentially result in changes in behaviour [30,31]. This is partially due to a lack of legislation or standards relating to the best practice for tree pruning and monitoring tree health and safety [19].

Morphological and physiological responses to root severance include a general reduction in overall vitality, premature leaf shedding and canopy dieback or reduced photosynthesis [32–34]. Little is known about the physiological effects of the pruning process [35] except those found in the studies of fruit trees in orchards [36,37]. The validity of cutting has been questioned for years as affecting the physiological balance of the tree [38]. Von Aufsess [39] noted the formation of a protective zone at the branch bases. Neely [40] observed that callus production (i.e., wound wood) at the margins of pruning wounds affected tree vigour. Gilman and Grabosky [41] found that the amount of discolouration and decay increased with the increasing size of branch to stem. Numerous studies have reported negative relationships between vitality and the presence of cavities [13,42–44]. Gruebler et al. [45] observed a correlation between the occurrence of decay cavities and the past removal of large main branches. Depending on tree species, pruning wounds of more than 5–10 cm diameter (up to 10 cm for trees species with good compartmentalisation potential and up to 5 cm for trees with poor capacity) were found to be a significant factor in discolouration, decay and cavities [25,46]. In turn, weakening the mechanical strength of the wood and increasing the hazard level. Sprouting is a well-recognised mode of regeneration where trees are cut down [47], but numerous studies [16,17,24,29,48–50] pointed out that recovered crowns may be expected to pose a risk to people and property.

Chlorophyll-a fluorescence (ChF) measurements, including the so-called JIP-test [51], are widely used in Eco physiological studies. They allow for the investigation of the physiological condition of the photosystem II (PSII) in chloroplasts [52]. The ChF measurements have been used to obtain a fast and non-invasive diagnostic method to detect damage to the photosynthetic apparatus of tree leaves in reaction to environmental stress [52–54]. The changes in the photosynthesis process can be caused by abiotic or biotic stresses and are possible to be found before the occurrence of visible symptoms of tree deterioration (leaf chlorosis, necrosis and growth limitation) [55,56]. Reported studies have confirmed its usefulness for the assessment of resistance to drought and other stress factors occurring in human-affected environments [57–60].

It was hypothesised that excessive crown cutting could lead to a shorter tree lifespan. Thus, this study aimed to study the impact of improper care practices on tree development based on their photosynthetic efficiency and the observation of regeneration processes. To our knowledge, **this is the first time that the chlorophyll fluorescence tool has been applied to estimate the effect of tree pruning on the photosynthesis process**.

## Materials and methods

### Site location

**Fig 1:**
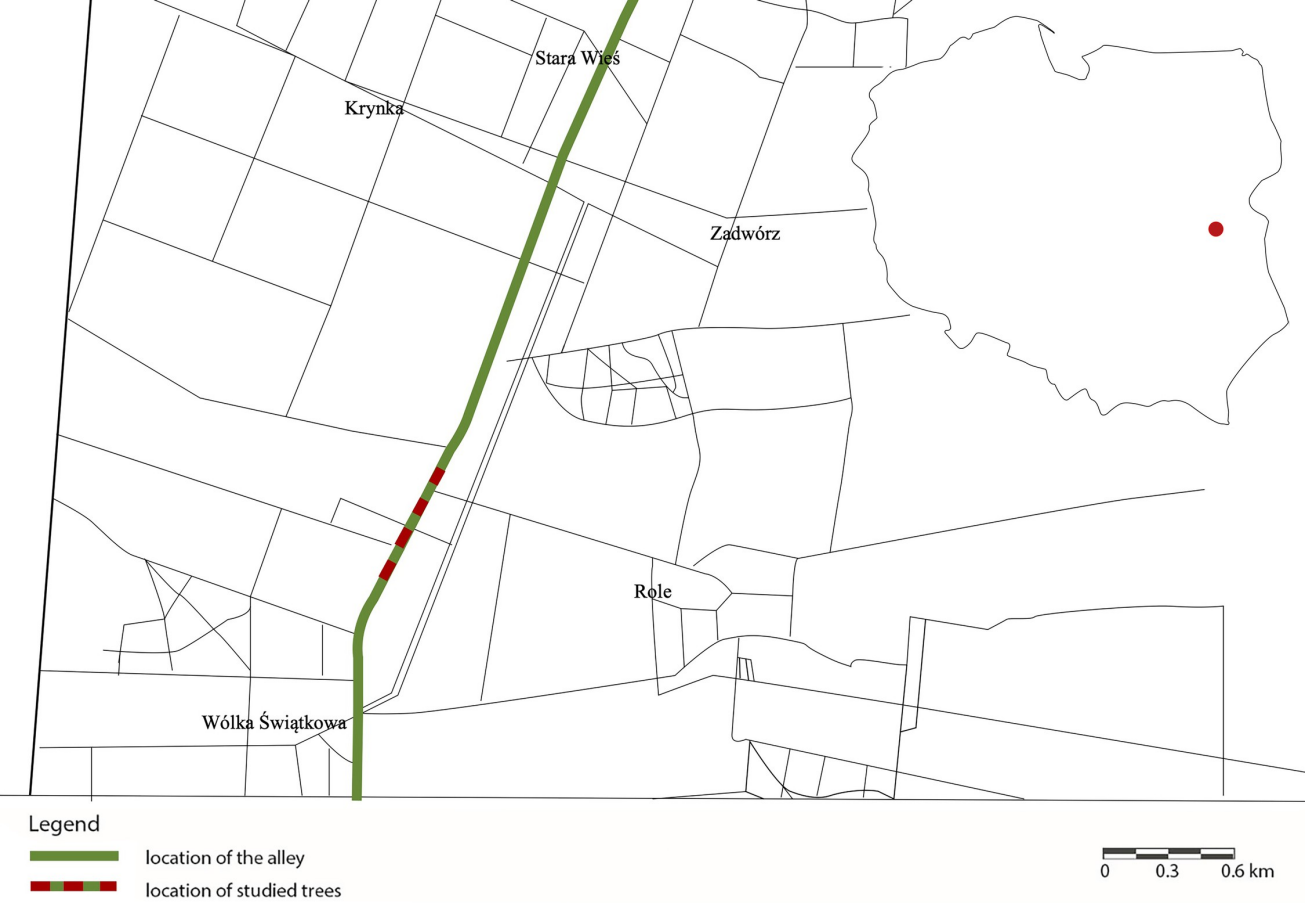
Location of the studied alley. With a read colour location of studied trees has been marked (source: Own elaboration).

The study was performed along an asphalt-paved rural road connecting Wólka Świątkowa and Krynka in the Lubelskie Province (51.9788˚N, 22.3935˚E, Fig 1). The road was planted with small-leaved lime trees (*Tilia cordata* Mill.), which formed an alley.

In September 2015, a severe pruning of 10 adjacent trees located on the eastern side of the road was carried out by the owner of the field adjacent to the road. The farmer claimed that the tree crowns were disturbing his farming activity. He removed the limbs with large diameters, minimum 40 cm; most trees lost one of the two codominant stems. Due to pruning, 50–60% of the tree crown volume was removed. This level of pruning severity is considered crown devastation. The trees growing on the opposite side of the road were left undisturbed and were treated as reference (control) trees. Besides crown pruning, no other damage or habitat stress factors were observed. The study included all the pruned trees (10 in total), **PT**, and ten undamaged trees growing opposite to PT and considered the control, **CT**. The total distance of the road where trees were growing was 4.2 km with partly irregular tree plantings. The 20 studied lime trees stand along a 300 m section of this road, on both sides (eastern and western), and they represented all the trees (CT and PT) growing within that road section. The distance between each tree ranges between 20–35 m. The tree line is approximately 12 m from the roadside. The average age of all studied trees PT and CT) was ca. 70 years. Measurements were carried out with the use of the Resistograph F 400 (IML, Germany).

### Biometric parameters

In July 2016 and July 2020, photographs of tree silhouettes were taken using the same observation point each time. The photographs were analysed in terms of the crown shape and sight-based tree crown projections. A diagram of canopy comparison was drawn using AutoCAD 2018 software (Autodesk). Each photograph was taken with a wooden 1 m long yardstick placed at the base of the tree trunk collar. The tree shapes of the trunks and crowns were scaled to their actual dimensions using AutoCAD software and traced. Then the outlines of each tree for 2016 and 2020 were placed on top of one another. This then allowed for a comparison of the crown areas. In October 2016, leaf samples were collected for the visual analysis and characteristics of shape and size anomalies.

### Phenological observations

Phenological observations were performed on 27 October 2016 to follow the differences in autumn leaf discolouration. Phenological stages were classified according to the BBCH scale [61]. The BBCH scale is applied to all growth stages of plants as its application relies on the description of the plant in the particular developmental time span [62,63]. In the analyses, the principal growth stage 9 was used to specify differences in phenological responses of the pruned and unpruned trees. This phase describes leaf senescence and the beginning of dormancy. It includes the secondary stages such as foliage still green and terminal buds developed (code 91), beginning of leaf discolouration (code 92), beginning of leaf fall (code 93), 50% of leaves fallen (code 95) and end of leaf fall (code 97).

### Chlorophyll-a fluorescence measurements

Chlorophyll-a fluorescence measurements were carried out on 1 August, 30 September, and 27 October 2016 on ten pruned and ten control trees. Twelve leaves were taken from each tree, with 120 samples at each date for damaged and 120 samples of control trees. The leaves were taken at a height of 2 m, 4 m, and 6 m, two leaves from each side: N, S, W, and E. Only a middle leaf was taken from each shoot. Samples were collected between 7 and 9 in the morning to ensure similar temperatures during leaf sampling. The leaves were packed into marked paper bags and transported in thermal bags for testing. Fast kinetics of ChF was measured using a *HandyPEA* fluorimeter (*Hansatech Instruments Ltd*., King’s Lynn, Norfolk, Great Britain) within 2 hours of sampling. The leaves were dark-adapted using light-excluding clips for at least 20 min. Two dark-excluding clips were applied to each leaf. The dark-adapted leaf samples were then illuminated with the 660 nm light at 3,500 μmol m^–2^ s^–1^. First, 120 samples from the control trees (CT) were tested, providing 240 records, then 120 samples from the damaged trees (PT) were tested, providing 240 records, which gave 480 records in total in August and the same set of data in September. Due to the scarcity of leaf samples, 240 PT record and only 173 CT records were taken in October.

The following 16 parameters were calculated from the ChF measurements: the density of active reaction centres (RC) of photosystem II per cross-section at point 0 (RC/CS_0_), the energy fluxes of average photon absorption (ABS), exciton trapping (TR), energy dissipation (DI), and electron transport (ET) per active RC (ABS/RC, TR_0_/RC, DI_0_/RC, ET_0_/RC), the maximum quantum yield of primary photochemistry (at t_0_), F_v_/F_m_ (TR_0_/ABS = φ_Po_), the probability that a trapped exciton moves an electron into the ETC beyond Q_A_, ET_0_/TR_0_ (ψ_Eo_), and both performance indices, PI_ABS_ and PI_total_. All these parameters are described in Table 1.

**Table 1:** Description of chlorophyll-a fluorescence parameters.

Fluorescence parameters	Description
F_0_ = ABS/CS_0_	initial fluorescence obtained from measurements also denoted as ABS/CS_0_
F_300_	fluorescence at 300 μs after illumination of a dark-adapted sample
F_2ms_	fluorescence at 2 ms after illumination of a dark-adapted sample
F_30ms_	fluorescence at 30 ms after illumination of a dark-adapted sample
F_m_	maximum fluorescence after illumination of a dark-adapted sample
V_K_ = (F_300_ ‒ F_0_)/(F_m_ ‒ F_0_)	relative variable fluorescence at 300 μs after illumination of a dark-adapted sample
V_J_ = (F_2ms_ ‒ F_0_)/(F_m_ ‒ F_0_)	relative variable fluorescence at 2 ms after illumination of a dark-adapted sample
V_I_ = (F_30ms_ ‒ F_0_)/(F_m_ ‒ F_0_)	relative variable fluorescence at 30 ms after illumination of a dark-adapted sample
ΔV_IP_ = 1—V_I_	the efficiency of electron transport from PSII to PSI
M_0_ = 4 (F_300_ ‒ F_0_)/(F_m_ ‒ F_0_)	approximated initial slope of the fluorescence transient, expressing the rate of RCs’ closure
F_v_/F_m_ = φ_Po_ = TR_0_/ABS = (F_m_ ‒ F_0_)/F_m_	maximum quantum yield of PSII photochemistry
ψ_o_ = ET_0_/TR_0_ = (F_m_−F_2ms_)/(F_m_−F_0_)	probability that a trapped exciton moves an electron into the electron transport chain beyond Q_A_,
δ_Ro_ = RE_0_/ET_0_ = (F_m_−F_30ms_)/(F_m_−F_0_)	probability that an electron from the intersystem electron carriers is transferred to reduce end electron acceptors at the PSI acceptor side
RC/ABS = γ_RC_/(1 –γ_RC_) = φ_Po_ (V_J_/M_0_)	Q_A_ reducing RCs per PSII antenna chlorophyll
RC/CS_0_ = φ_Po_ (V_J_/M_0_) (ABS/CS_0_)	density of active RCs (.Q_A_ reducing RCs) per cross-sectioncross section at point 0
PI_ABS_ = RC/ABS × φ_Po_/(1 –φ_Po_) × ψ_Eo_/(1 –ψ_Eo_)	performance index (potential) for energy conservation from photons absorbed by PSII to the reduction of intersystem electron acceptors
PI_total_ = RC/ABS × φ_Po_/(1 –φ_Po_) × ψ_Eo_/(1 –ψ_Eo_) × δ_Ro_/(1 –δ_Ro_)	performance index (potential) for energy conservation from photons absorbed by PSII to the reduction of PSI end electron acceptors

### Tree vitality

According to Roloff’s classification, the vitality assessment of each tree was performed [64] based on leaf and branch growth pattern. The condition of each tree was evaluated based on the liveliness of the distal crown parts. Trees were divided into four groups: R0 ‘exploration’: trees in the phase of intensive offshoot growth, R1 ‘degeneration’: trees with slightly delayed offshoot growth, R2 ‘stagnation’: trees with visibly delayed offshoot growth, R3 ‘resignation’: trees without regeneration possibility nor returning to second class.

### Safety evaluation

According to the VTA methodology (*Visual Tree Assessment*), risk evaluation was used for the assessment of damaged and control trees. Trees were examined by visual evaluation and segregated into one of the five risk classes: insignificant (A), low (B), moderate (C), high (CD), extreme (D). According to the quantitative typology formulated by ISA (*International Society of Arboriculture*), safety measures were performed to the standard VTA safety procedure [65]. To evaluate a tree’s risk, the VTA method considers such factors as assessment of decay extent in trees and tree structural defects, exposition to the wind, occupancy, evaluation of the target, site consideration, and damage to the root system [66–68]. A tree risk assessment decision depends on the expert knowledge and experience and can be influenced by risk perception, risk acceptance, and professional bias of an expert [69].

### Statistical analysis

Biometric parameters: because the data was not normally distributed (according to the Shapiro-Wilk test), the non-parametric Mann-Whitney U-test was performed. To find the differences between RT and CT Chlorophyll fluorescence: the data showed normal distribution (according to the Shapiro-Wilk test); therefore, Student’s *t*-test was used. All calculations were made using *STATISTICA version 13*.*0* software (*TIBCO Software Inc*. *(2017)*, http://statistica.io., USA).

## Results

### Biometric parameters

**Fig 2:**
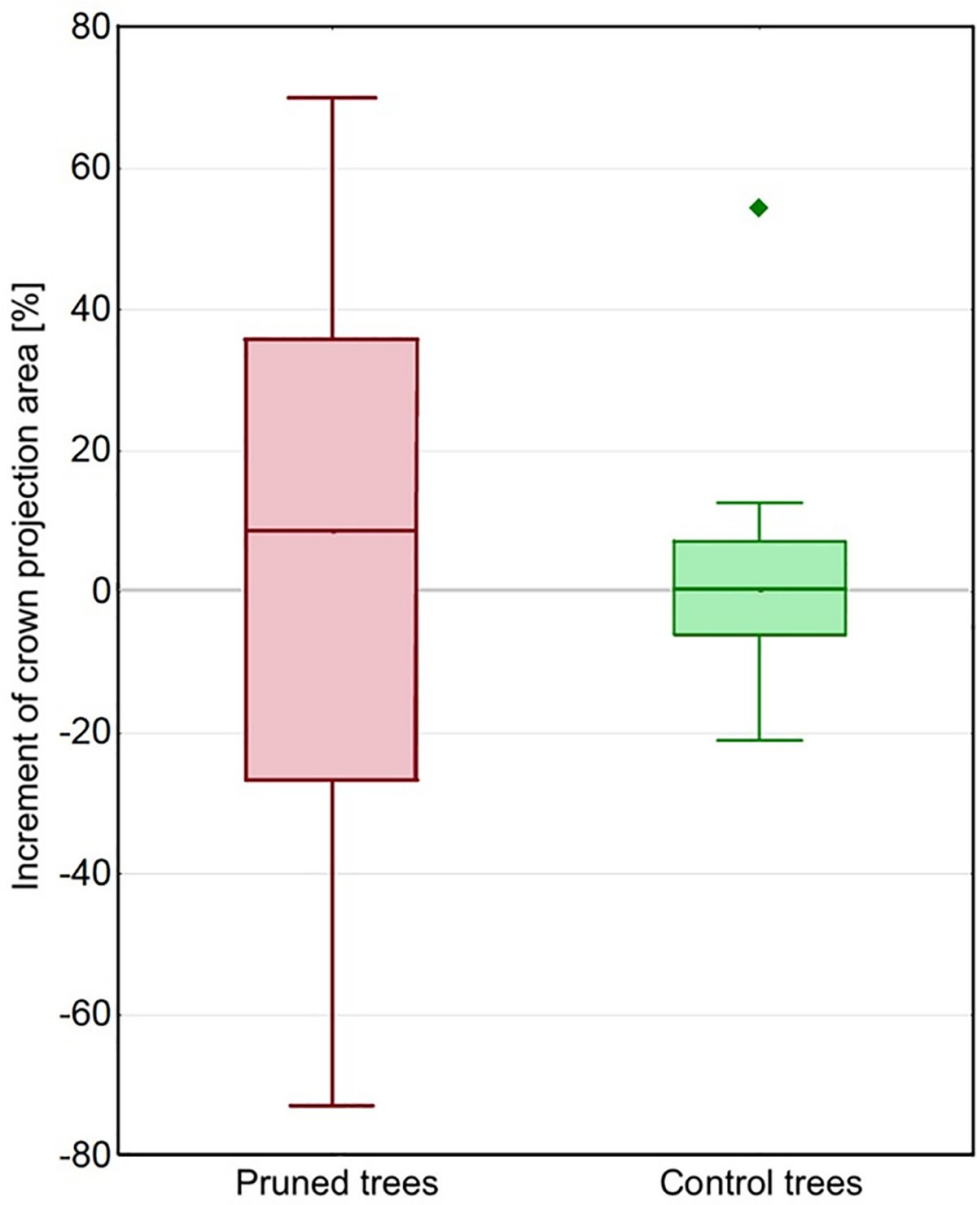
Increment [%] of the area of sight-based tree crown projections; medians, whiskers indicate variability outside the upper and lower quartiles.

**Fig 3:**
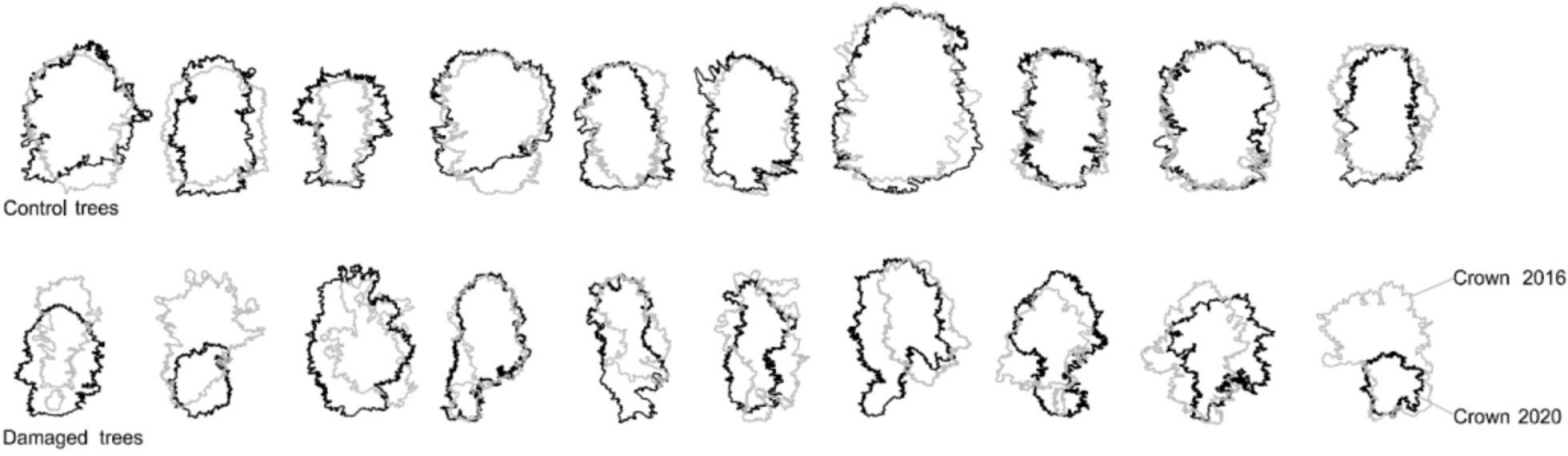
Comparison of tree silhouettes, 2016 and 2020.

Control trees demonstrated weak crown growth with a median of 0.49%, p = 0.960 (Fig 2). Most of the pruned trees reconstructed their crowns, with a median of 8.767%; however, specimens with an apparent regression in crown volume were recorded, *i*.*e*. trees No. 2 (-58.64%) and 10 (-72.96%). The trunks of those two specimens were broken due to extensive decay developed in the area of post-cutting wounds. According to the results, the stress had a devastating effect on PT. To clarify the pattern of changes, a visual comparison of the particular PT and CT crown shapes from 2016 and 2020 provides a good demonstrational model (Fig 3).

### Leaf health status evaluation and phenology

**Fig 4:**
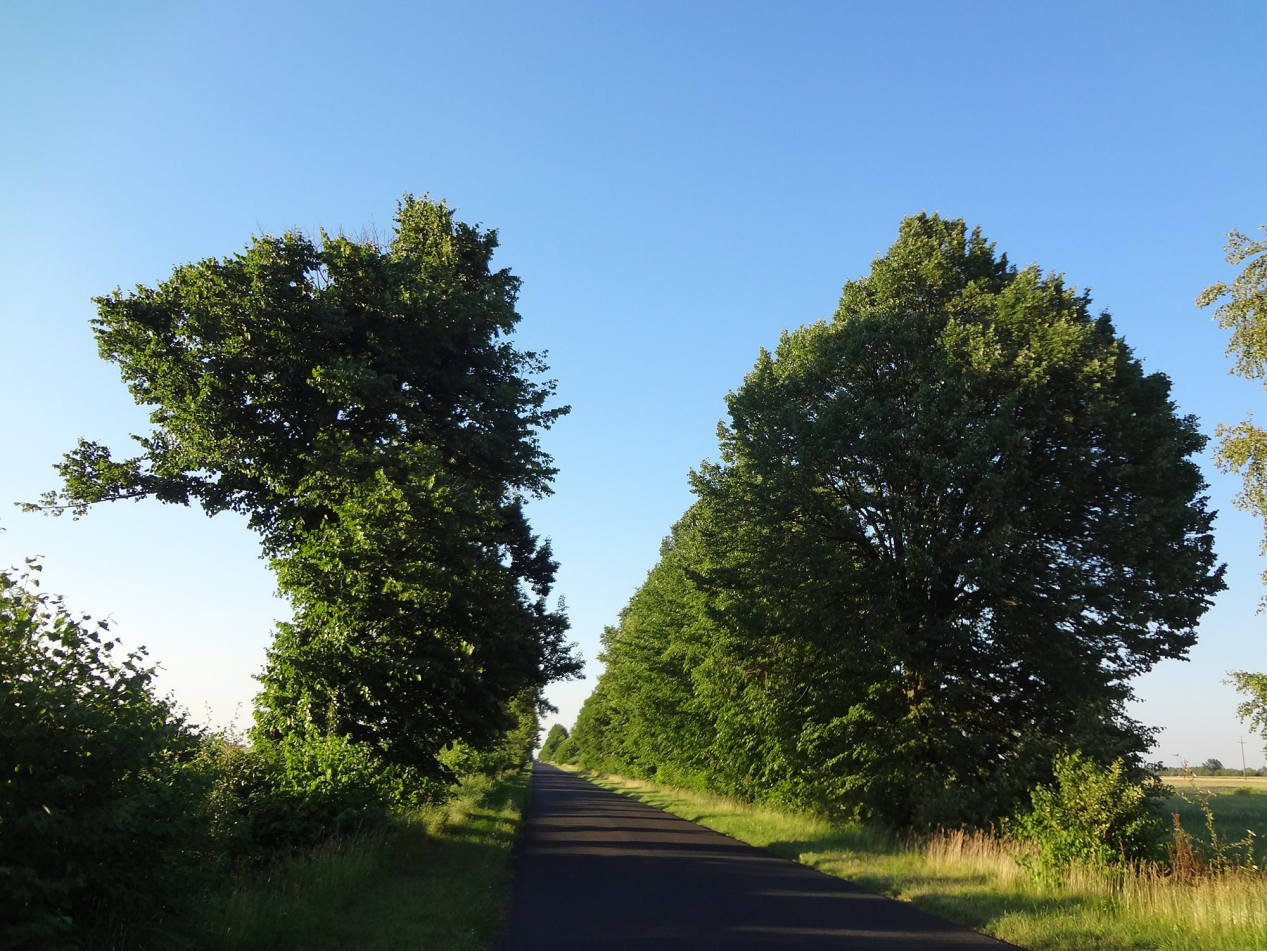
Crowns of damaged trees (left) and control trees (right), June 18, 2016. Photo by M. Suchocka.

**Fig 5:**
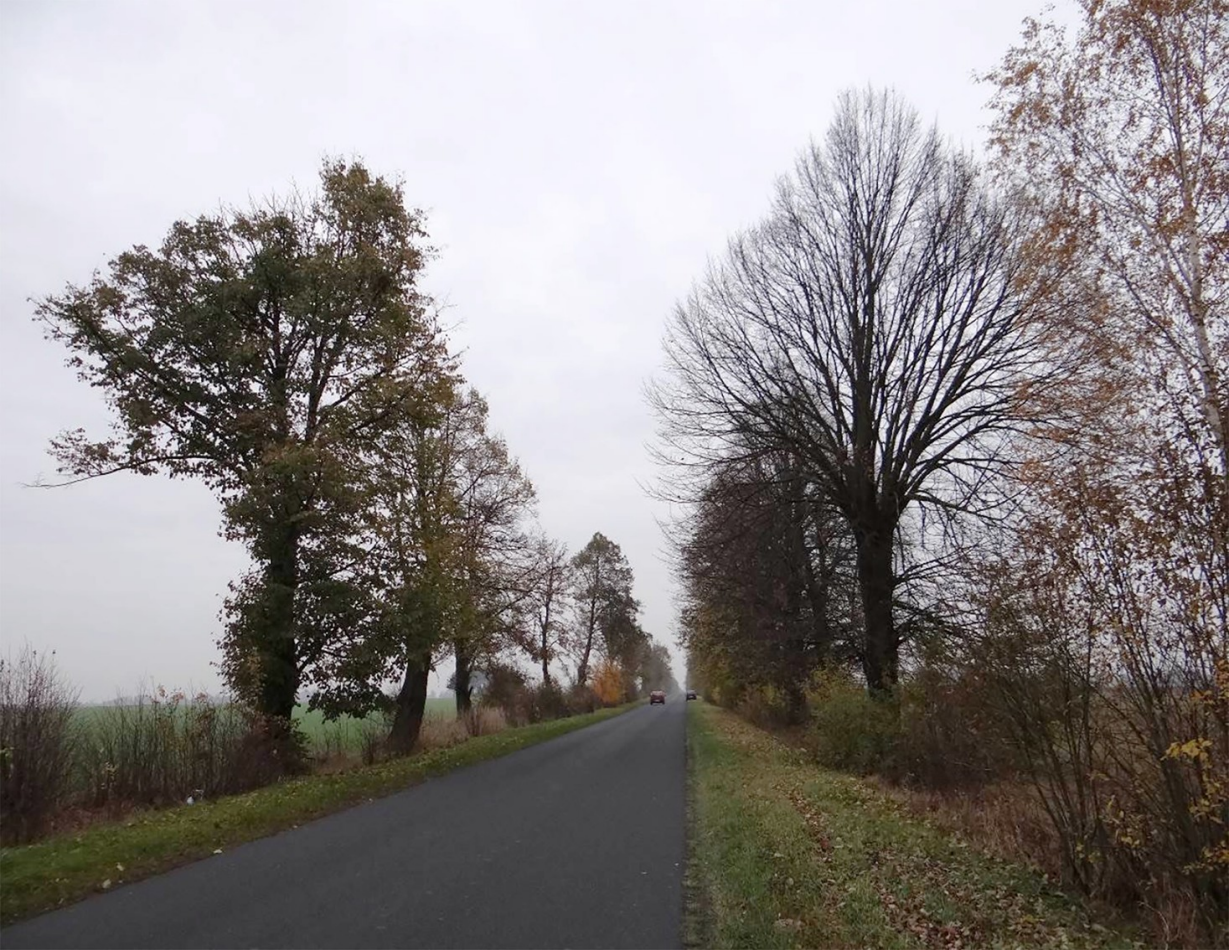
Crowns of damaged trees (left) and control trees (right), October 27, 2016. Photo by M. Suchocka.

In June 2016, all trees showed a similar pattern of leaf development (Fig 4). The CT leaves retained full vitality from June to September. During the growing season of 2016, PT had rebuilt their crowns from the remaining limbs and numerous shoots developed intensively from the epicormic buds. In those trees, leaf vitality was observed until late October (Fig 5).

**Fig 6:**
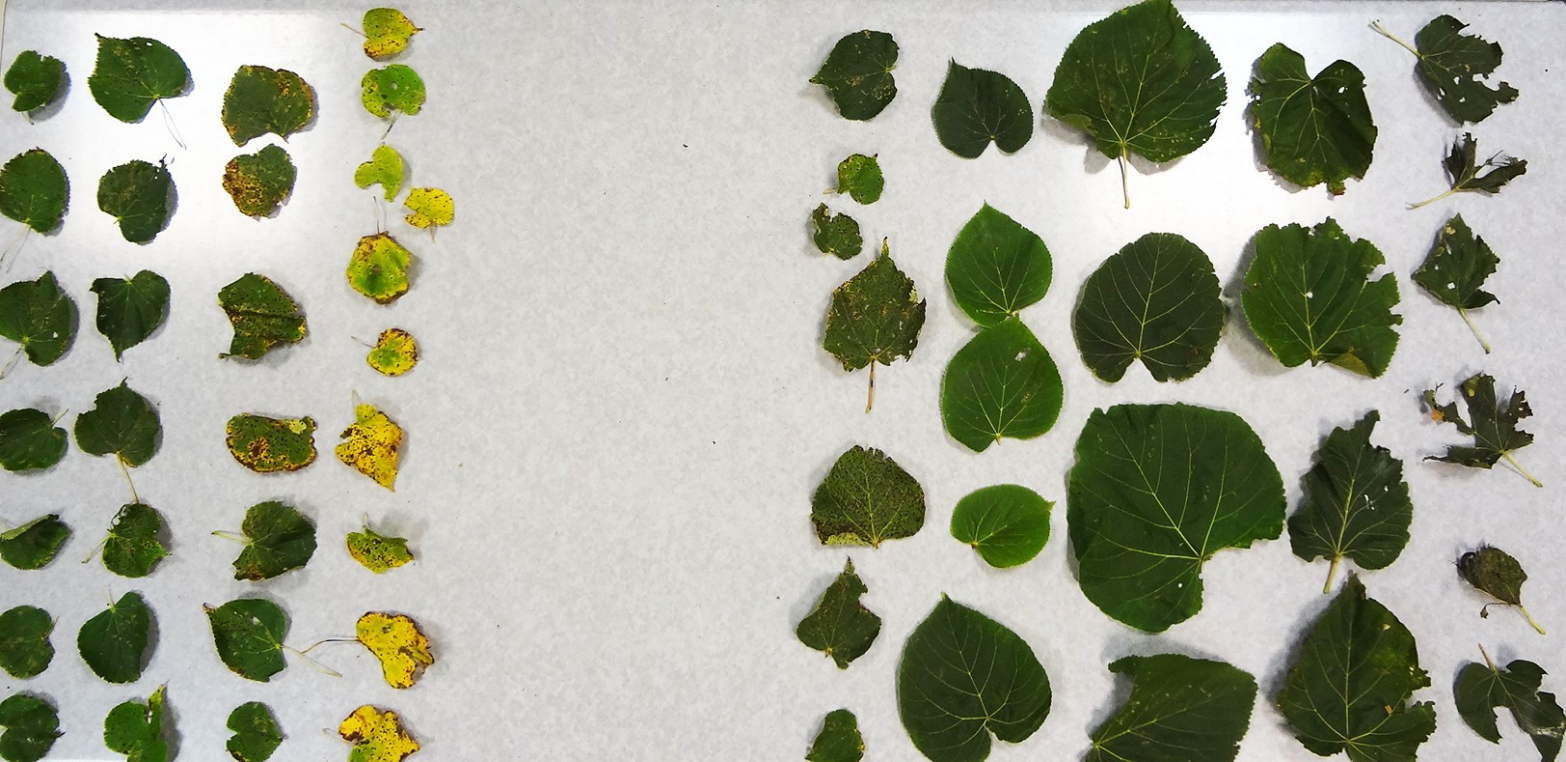
Leaves collected from CT, on the left, leaves from PT from within the offshoots’ zone, on the right, September 30, 2016. Photo by M. Suchocka.

During the hot and dry summer of 2016, CT developed visually smaller leaves. Leaves of PT were larger, particularly within the offshoots’ zones. We have found up to a fourfold difference between the diameter of the smallest CT and largest PT leaves and up to double the diameter between typical CT and the biggest PT leaf. The differences in the health status of the leaves in CT and PT were visually detected on 30 September. The leaves in PT were still green while in CT first leaves showed autumn discolouration. The offshoot leaves were oversized and thinner than leaves developed on typical shoots. As they were not resistant to wind, most of the leaves were damaged (Fig 6). By 27 October, 80% of CT leaves had fallen, while in PT, a significant portion of leaves remained green, especially in the offshoots zone.

**Fig 7:**
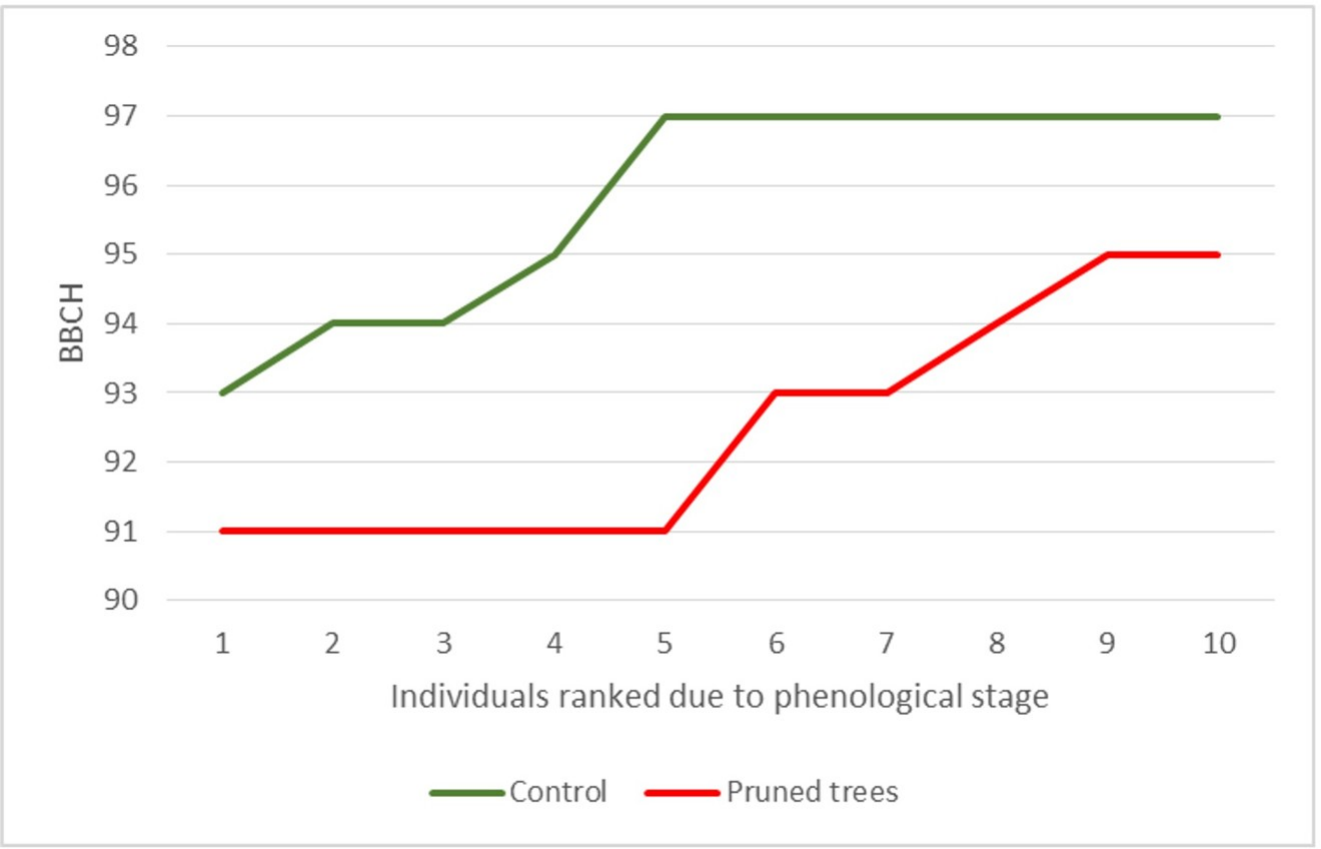
Progression in phenological stages in control and pruned trees on October 27, 2016.

In autumn, PT showed delayed phenological stages compared to CT. On 27 October 2016, all (or almost all) leaves fell from six of the trees and the early autumn phenological stages were observed only in the remaining four individuals. By contrast, five pruned trees still had fully green foliage and another five individuals showed up to 50% of leaf fall or leaf discolouration only (Fig 7).

### Chlorophyll a fluorescence (ChF) technique

**Fig 8:**
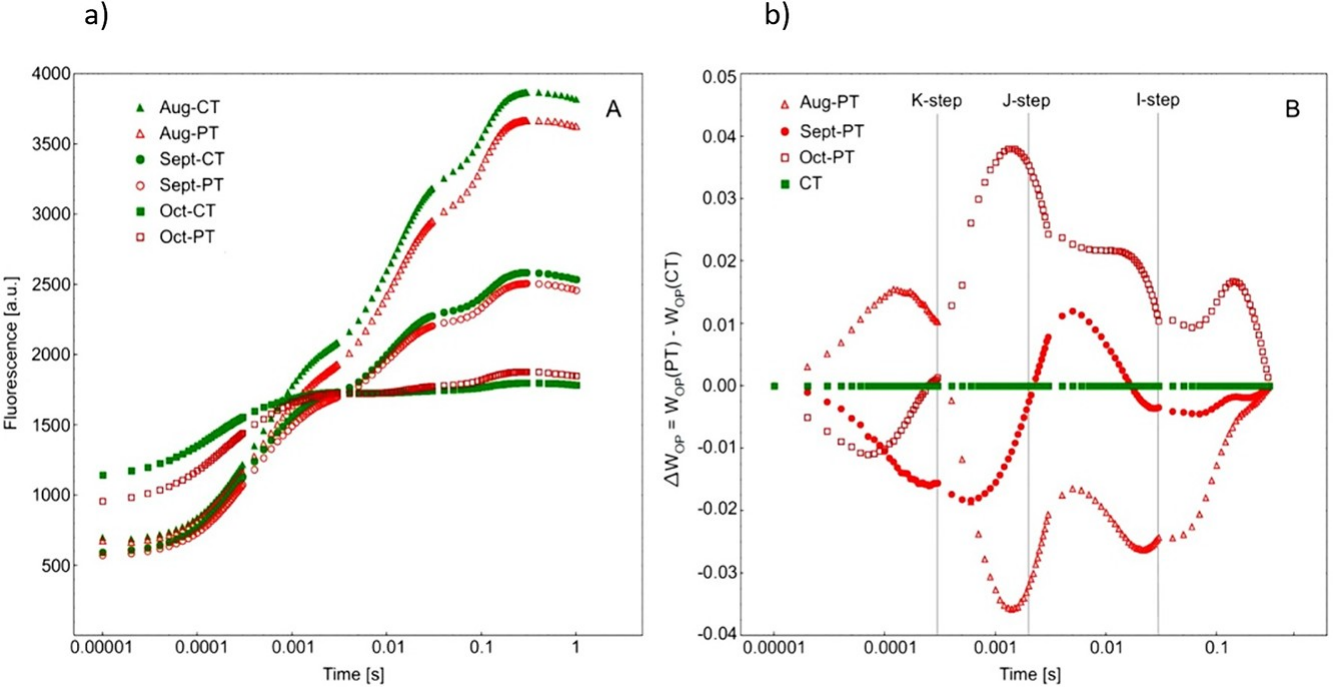
Chlorophyll fluorescence induction curves (a) and double-normalised plots of ChF induction curves (b) of leaf samples taken from pruned (PT) and control (CT) trees on August 1, September 30, and October 27, 2016.

Fast chlorophyll fluorescence induction curves and their double-normalised plots showed minor differences between PT and CT, indicating that the performance of photosynthetic apparatus in pruned trees was balanced to some extent (Fig 8). In summer, PT leaves revealed some disturbances at K-step (0.3 ms), while at J-step and I-step the fluorescence was lower. In contrast to CT, in late autumn the fluorescence at J-step and I-step in PT was higher. Nevertheless, the discrepancies were not high, up to 4% of the control values.

**Fig 9:**
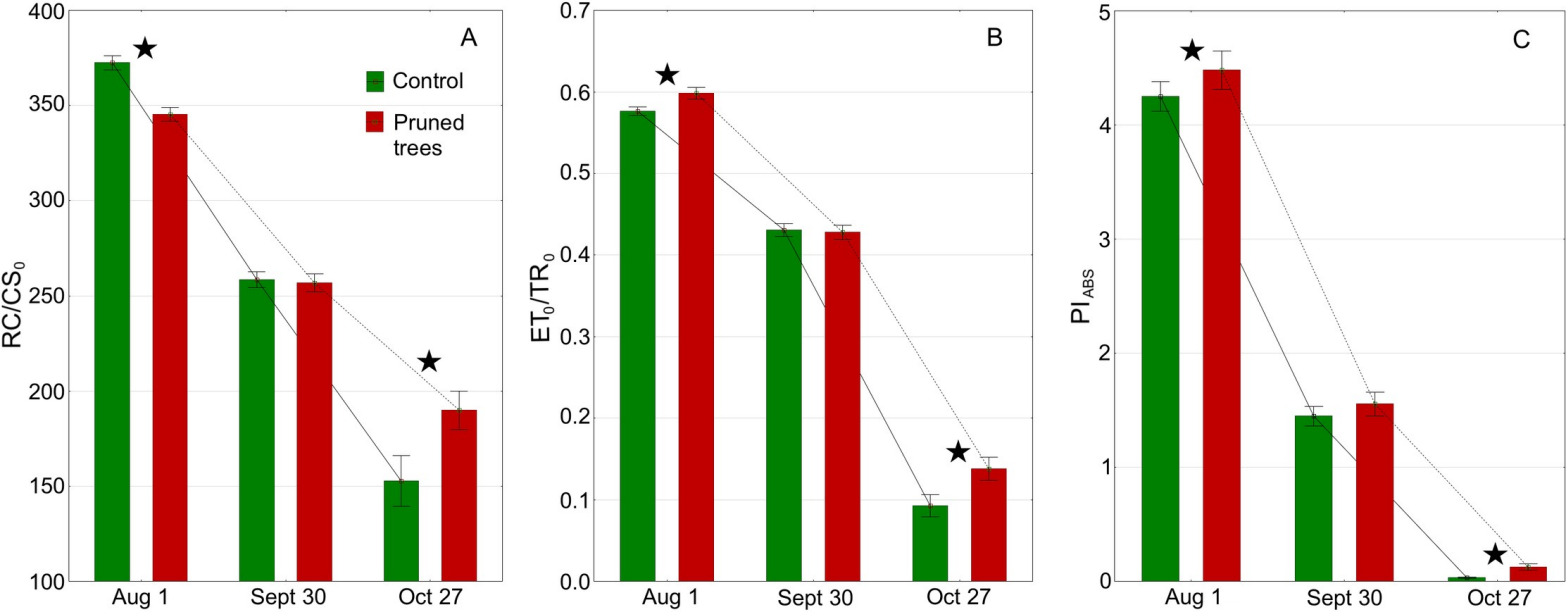
Changes in density of reaction centres, RC/CS_0_ (a), probability of electron movement beyond Q_A_, ET_0_/TR_0_, (b), and performance index on absorption basis, PI_ABS_ (c), in pruned (PT) and control (CT) trees during the first growing season after pruning. Means; whiskers and asterisks indicate SE and statistical significance, respectively.

The most favourable values of RC/CS_0_, F_v_/F_m_, ET_0_/TR_0_, PI_ABS_, and ET_0_/RC were obtained in August and decreased over time, whereas ABS/RC, TR_0_/RC, and DI_0_/RC increased (Table 2 and Fig 9). During the summer, the PT leaves revealed a significantly lower (*p* = 0.0000) density of reaction centres, RC/CS_0_ was 258.4 and 372.5 in PT and CT, respectively. In late October, RC/CS_0_ in CT diminished to a greater extent than in PT, *p* = 0.0000 (Fig 9A). The maximum quantum yield of primary photochemistry (F_v_/F_m_) was favourable in both groups in August however, it was significantly higher in CT (Table 2). Towards the end of October, F_v_/F_m_ decreased in both CT and PT, showing significantly lower values in CT. The calculated parameter ET_0_/TR_0_ was significantly higher in PT both in August and October (Fig 9B), likewise ET_0_/RC, while in late September it was significantly higher in CT. During the summer, ABS/RC, TR_0_/RC, and DI_0_/RC were significantly higher in the pruned trees, whereas in late October, these parameters were higher in the control trees. The huge increase in ABS/RC and DI_0_/RC was observed at the end of the growing season, indicating severe constraints in the pool of electron carriers.

**Table 2:** Chlorophyll fluorescence parameters in undamaged (control) and severely pruned trees recorded in the first season after pruning.

		F_v_/F_m_			ΔV_IP_			ET_0_/RC	
Date	Control	Pruned	*p*	Control	Pruned	*p*	Control	Pruned	*p*
Aug 1	0.837	0.833	0.0019	0.212	0.237	0.0000	0.819	0.883	0.0000
Sept 30	0.779	0.775	0.4544	0.152	0.152	0.8956	0.719	0.684	0.0000
Oct 27	0.328	0.458	0.0000	0.168	0.111	0.0019	0.203	0.273	0.0002
		**ABS/RC**			**TR**_**0**_ **/RC**			**DI**_**0**_ **/RC**	
**Date**	**Control**	**Pruned**	***p***	**Control**	**Pruned**	***p***	**Control**	**Pruned**	***p***
Aug 1	1.703	1.786	0.0000	1.424	1.484	0.0000	0.279	0.302	0.0000
Sept 30	2.174	2.124	0.1587	1.680	1.616	0.0000	0.494	0.507	0.5976
Oct 27	23.869	8.885	0.0013	2.403	2.224	0.0000	21.466	6.661	0.0014

During summertime, the Pl_ABS_ parameter was the highest, 4.25 and 4.48 in CT and PT, respectively, and decreased towards the end of the growing season reaching 0.03 (CT) and 0.125 (PT) in late October (Fig 9C). The differences between CT and PT were significant in August and October, 0.035 and 0.000, respectively, but not in September. In October the transients both in PT and CT were visibly flattened. The calculated parameter δ_Ro_ showed confusing values; therefore we did not analyse PT, with *p* = 0.0017 and *p* = 0.0125, respectively (data not shown). Differences in ΔV_IP_ in PI_total_ between CT and PT were significant in August and October, with higher and lower values in PT, respectively.

### Tree vitality

In 2016, the vitality of control trees was defined as phase R0 ‘exploration’ or R1 ‘degeneration’ according to Roloff’s classification. Pruned trees were classified as R2 (50%) and R3 (50%). In 2020, only one control tree was defined as R2 whereas other trees were still at the R0 phase. Crowns of pruned trees showed different vitality classes: R1 (one tree), R0/1 (one tree), R1/2 (7 trees) and R2 (only one tree), mainly because of epicormic sprouting in newly built parts of the crowns (Tables 3 and 4).

**Table 3:** Tree vitality, safety evaluation and detailed characteristic of wounds in pruned trees.

Lp.	Latin name	Roloff 2016	Roloff 2020	Risk class 2016	Risk class 2020	Comments 2020
1.	*Tilia cordata*	2	0/1	B	C	The fruiting body of *Phellinus ignarius* on the trunk of the wound. Monitoring required in 3 years.
2.	*Tilia cordata*	2	2	B	D	Reduced crown (broken in half), extensive stem necrosis, *Ganoderma lucidum* at the base of the stem. Necessary removal of a tree due to the risk.
3.	*Tilia cordata*	3	1/2	B	C	A decaying cavity at a height of 2 m. Necessary monitoring in 3 years.
4.	*Tilia cordata*	3	1/2	B	C	Wound with necrosis after removed codominant trunk. Monitoring required in 3 years.
5.	*Tilia cordata*	2	1/2	B	CD	Small leaves, visible dead branches in the crown. At the crown collar, in the vicinity of the wound, the fruiting body of *Polyporus squamosus*, in the crown dieback. On the trunk visible large necrosis. Necessary monitoring next year.
6.	*Tilia cordata*	3	1/2	B	CD	Dieback, wounds with necrosis on the trunk at a height of 3 m. Necessary monitoring next year.
7.	*Tilia cordata*	2	1/2	B	C	Wounds with necrosis on the trunk. Necessary monitoring in 3 years.
8.	*Tilia cordata*	3	1/2	B	C	Wounds with necrosis on the trunk. Necessary monitoring in 3 years.
9.	*Tilia cordata*	2	1/2	B	CD	Wounds, necrosis caused by the sun, *Polyporus squamosus*. Dying and dry tops, branches, about 20% of the crown, Monitoring necessary next year.
10.	*Tilia cordata*	3	1	B	CD	Wounds, dry branches and dieback, reduced tree height. Necessary monitoring next year.

**Table 4:** Tree vitality, safety evaluation and detailed characteristic of wounds in control trees.

Lp.	Latin name	Roloff 2016	Roloff 2020	Risk class 2016	Risk class 2020	Comments 2020
1.	*Tilia cordata*	0	0	B	B	
2.	*Tilia cordata*	0	0	B	B	
3.	*Tilia cordata*	1	0	B	B	
4.	*Tilia cordata*	0	0	B	B	
5.	*Tilia cordata*	0	0	B	B	
6.	*Tilia cordata*	0	0	B	B	
7.	*Tilia cordata*	1	0	B	B	
8.	*Tilia cordata*	0	0	B	B	
9.	*Tilia cordata*	0	0/1	B	CD	Dieback. Necessary monitoring next year.
10.	*Tilia cordata*	0	2	B	B	

### Safety evaluation

According to the VTA methodology, the risk class of control trees was assessed at B level (low risk) in 2017 and most of them in 2020. In 2020 only one CT (No. 9), was classified at CD level (high risk). That tree required only dead branches removing and monitoring.

All PT were classified at B level (low risk) in 2017, but the assessment in 2020 showed numerous structural problems. Four years after pruning many signs of weakened statics were found, evidence of decay marked by cavity-nesting holes or fungal conks (*Ganoderma applanatum* (Pers.) Pat., *Phellinus ignarius* (L.) Quél., *Polyporus squamosus* (Huds.) Quél. Two PT trunks were broken and a significantly reduced height was observed (Tables 3 and 4).

Photographs of PT and CT were taken in 2016 and 2020 are included in the S1 Table. The images show the crown shape of the trees in 2016 and 2020 and the risk-increasing traits on branches and trunks caused by wounds coming from the crown reduction. All observed wounds had diameters in the range of 10–60 cm and initiated hollows and necrosis formation on the bark of the trunks. Conks of aggressive parasitic fungi, developing in the wounds after cutting, were found in some PT.

## Discussion

Roadside trees in typical situations face a widely discussed problem of high mortality caused by stress factors such as air and soil drought, soil contamination and compaction [56,70–72]. The roadside trees in this study developed without constraints on the root system in a wide roadside with open space. The tree trunks were not damaged in any way. The only stress factor, in this case, was intensive cutting within the tree crowns.

Pruning is one of the most common services. Pruning impacts both tree health and structure [17] and could be one of the best and one of the worst things that can be done to a tree [73]. There is no research to suggest that crown thinning improves either tree health or structural stability. Grabosky and Gilman [74] and Dujesifken [75] discovered that discoloured areas in wood increased with the amount of pruning and the capacity to heal wounds decreased at the same time. Clark and Matheny [17] showed that lifting the height of crown base in the 6- to 8-year-old plantation Monterey pine in Tasmania (Australia) to 45% of the tree height did not affect its growth. These results suggest maintaining a live crown ratio of 55%. Slabaugh [76] and Neilsen and Pinkard [77] found that growth was not adversely affected by the removal of up to 50% of the live crown of young trees by lifting, as shown by the results of eight field studies with Douglas-fir (*Pseudotsuga menziesii*). On the other hand, Borowski and Pstrągowska [78] showed limited trunk radial increments in European ash after severe pruning. O’Hara [79] suggested a 33% crown removal limit. Kosmala et al. [14] defined the critical level for tree vitality at 55% of branches and twigs in case of mechanical damage. Most of the authors determined the critical values (levels) at which damaged trees die in a shorter or longer time as 50–55%. In all studied trees, more than 50% of the crowns were removed.

Crown damage is considered to be less important than root and trunk wounding [13,80]. This attitude is probably a result of a limited number of studies on crown damage. Koch’s method [13] introduced the differentiation between crown parts as a subject for evaluation. Damage to the area of little twigs is considered to be of less importance, damage to thin branch zone is not taken into consideration in case of well-regenerating tree species, while the zone of main branches and trunk base is evaluated in every case since it is crucial to the tree’s overall condition. In the case of this study, main branches were cut on the crown collar with a serious impact on the structure of the tree.

In this study PT showed deformed silhouettes, intensive epicormic bud sprouting, and extreme differences in crown growth reduction from 69.85% to 72.96% (standard deviation 46.18%). The CT showed a standard deviation of 20.21%. The purpose of epicormic bud appearance is to rebuild the crown in response to damage or physiological inefficiency [81]. Fini et al. [35] observed root suckers released by 70% of topped trees during the growing season after pruning. Buds formed close to the crown are ready to grow in the case of crown damage [82]. In PT, intensive sprouting was observed over all parts of the damaged trees, from the trunk collar to the end of the remaining part of the crown. In the case of CT, no sprouts developed at the same time. Moreover, Fini et al. [35] observed the increased presence of dieback on pruned branches due to topping. We found numerous diebacks on the top of the cut trees, typical for ancient trees. In general, our results confirm the findings of Fini [35] that apical control and apical dominance are key issues for the preservation of a structurally sound tree structure. Factors such as the volume of leaf area removed have a less important impact on the morpho-physiological response to pruning and the long-term efficiency of the photosynthetic apparatus.

Epicormic sprouting is a response to stressors [83,84] e.g. related to crown loss. Insect defoliation [85], fire [84], frost [86], wind damage [87] are the features that promote either re-establishing of the leaf area following a disturbance or maintaining the functional and effective crown [88,89]. Following a severe stress event, such as a top removal, trees initiate a stress response that stimulates the growth of meristems to replace lost biomass rapidly. Even species that typically do not form sprouts maintain sprouting capacity in the situation of severe disturbance [83].

The sprouting capacity may be explained as compensatory growth ability due to the “coordination theory” [90]. If the shoot/root ratio is altered by pruning, the intensified leafy shoot growth is observed. In our investigation, some severely pruned trees recovered their crowns within four years (however, a reduction was also observed in particular specimens). The intensified shoot growth was accompanied by the development of enlarged leaf blades. Leaf size response to pruning is commonly observed in horticultural and arboricultural practice. Severely pruned or damaged trees and shrubs often develop abnormally large leaf blades in the same growing season [35]. Moreover, our analysis showed that leaf senescence in pruned trees was delayed. A similar delay in leaf phenology stages was observed on London plane (*Platanus ×hispanica* ‘‘Acerifolia’) roadside trees severely affected by salt deposition on shoots along roads with heavy traffic [91].

Chlorophyll-a fluorescence analysis shows photosynthetic performance inside and around photosystems II of examined leaf sample and enables the detection of any alterations caused by stress factors [92]. To our knowledge, up-to-date chlorophyll fluorescence measurements, particularly JIP-test analysis, have not been applied in research related to tree pruning. Some experiments were conducted to measure leaf CO_2_ assimilation and other gas exchange parameters in trees. Li et al. [93] found that summer pruning in apple trees reduced the whole canopy carbon fixation. On the other hand, increased net CO_2_ assimilation in individual leaves was observed in partially defoliated or pruned trees [94–97]. Pinkard et al. [96] showed that regardless of the increased C assimilation in newly produced ramets, C assimilation in severely pruned young *Eucalyptus nitens* trees was amplified by the increased severity of pruning. Hipps et al. [97] found that oversized leaves developed from the regrown shoots in the London plane had higher C assimilation and stomatal conductance (g_s_). Likewise, Fini et al. [35] demonstrated reduced both stomatal and non-stomatal limitations to CO_2_ assimilation in leaves developed after pruning. Hipps et al. [97] explained those findings as an effect of higher exposition of leaves to light. Other authors indicated that increased photosynthetic activity in new sprouts and leaves developed following damage is a tree’s response to maintaining crown productivity [82,98].

The leaves from both control and pruned trees were collected from the same position on shoots (the middle-positioned leaves), which are usually exposed to light due to their scattered arrangement around the shoot. In the experiment, the leaf samples were collected from each side of the crown, i.e. from N, E, S, and W position. Thus, it can be assumed that the averaged ChF parameters in leaf samples collected from around the crown are representative of the general trends in each examined group. In PT some new shoots emerging from damaged branches were included according to the adopted method of sample collection. However, according to observations, they were no more exposed to better or worse light conditions than the leaves from undamaged branches. The chlorophyll-a fluorescence technique provides numerous parameters and many of them are sensitive to light [51,99,100]. Leaf position within the crown may affect light availability for mesophyll cells. In tree stands, lower leaves, if shaded, may be more susceptible to changing light conditions than upper and medium leaves [101]. Moreover, restricted light availability may decrease efficiency/probability parameters, e.g. F_v_/F_m_, ET_0_/TR_0_, and PI_ABS_ [99]. On the other hand, upper leaves may show diminished photosynthetic capacity due to higher irradiance [102]. The trees in our examination grow along the road and separated from each other, thus, their crowns are not shaded along a vertical gradient. There was no difference in the maximum quantum efficiency of PSII (F_v_/F_m_) between pruned and control trees in September. In August F_v_/F_m_ was significantly higher in (Table 2), however, the values exceeding 0.83 suggest optimum PSII efficiency. This is consistent with the findings of Zivcak et al. [100] who ascertained no sensitivity of F_v_/F_m_ to changing light conditions. The efficiency of electron movement into the electron transport chain beyond Q_A_, ET_0_/TR_0_, and both performance indices, PI_ABS_, and PI_total_, were higher in damaged/regrowing trees both in summer and in late autumn, which may suggest the effect of better light availability [100]. However, considering the pattern of our results the shifted photosynthetic capacity was caused by organs’ regeneration rather than the effect of light conditions. The CO_2_ assimilation pattern often follows PSII vitality detected by chlorophyll fluorescence [95,103]. A higher photosynthetic capacity of photosystems undoubtedly provides more energy for C assimilation. Thus, in our opinion, the photosynthetic apparatus in the damaged/re-growing trees was better adjusted to increased demands of assimilates and the photosynthetic apparatus capacity was maintained for a longer time to provide so-called “compensatory photosynthesis” [35]. Maurin and DesRochers [104] found that pruning increased in the case of 2/3 of the live crown pruned poplar trees, net photosynthesis of residual foliage and nitrogen foliar concentrations but reduced total non-structural carbohydrates reserves of roots. Two growing seasons after pruning trees reduced both breast height diameter and height growth. Moreover, compensatory photosynthesis should be proportional to the amount of foliage removed Hart et al. [105] showed that stomatal conductance of residual leaves increased with defoliation up to a certain point, after which it was similar in 50% and 98% defoliated aspen trees.

Yordanov et al. [106] showed that partial decapitation and defoliation increases F_v_/F_m_ (denoted as φ_Po_), ET_0_/TR_0_ (denoted as ψ_Eo_), PI_ABS_ and RC/ABS in *Phaseolus vulgaris*. This paper is likely the first report about the effect of severe pruning in trees on OJIP parameters. Most studies concerning trees or other woody plants underline the increased CO_2_ assimilation as a result of pruning [30,104,107,108]. Ovaska [107] and Turnbull [108] showed that higher CO_2_ assimilation is related to higher Rubisco activity rather than to N availability or even stomatal conductance. It seems that due to the increased demand for sugars the activity of both photosystems I and II was increased to accelerate linear photosynthetic electron flow in the leaves of heavily pruned trees. The multi-parametric Performance Index, PI_ABS_, showed a visible increase both in August and October. It should be noted that PI_ABS_ depends not only on F_v_/F_m_ but also on ET_0_/TR_0_ and RC/ABS. Increased ET_0_/TR_0_ in August contributed to higher PI_ABS_ in pruned trees despite lower F_v_/F_m_. Likewise, IP-phase was positively affected in pruned trees in August showing an enhanced probability of electron transport around PSI.

In August and September ChF transients showed a typical pattern with two intermediate steps, J and I. In October the transients both from pruned and control trees were visibly flattened. The measurement results and some calculated OJIP parameters of numerous samples were confusing. Due to statistical rules, such records should be rejected. Nevertheless, we decided to include those records to illustrate the senescence progress which was not parallel in pruned and control trees. Indeed, the senescence in pruned trees was visibly delayed (Fig 7). Particularly, the October data necessary for PI_total_ calculation was confusing and was not analysed. Therefore for October, we analysed only ΔV_IP_, a comprehensive parameter indicating the efficiency of electron transport from PSII to PSI, instead of PI_total_. In pruned trees, ΔV_IP_ was the highest in August and the lowest in October. A high probability of more efficient electron transport towards end electron acceptors in PSI was found in *Fagus sylvatica* trees of provenances growing under continuous stress conditions [108]. Additional stresses, such as water deficit did not affect ΔV_IP_ in those hardened populations. On the contrary Pšidová, et al. [109] ascertained a higher difference in ΔV_IP_ between individuals affected and non-affected by drought stress in the case of trees coming from less stressful habitats. In our examination, oversized leaves from pruned trees showed a higher difference in ΔV_IP_ between summer and autumn measurements, while in control trees the seasonal changes were not so visibly pronounced (Table 2). This suggests that leaves developed on pruned trees may reveal higher plasticity or, on the contrary, they are more susceptible to changing abiotic conditions.

During the growing season, the number of active RCs in leaf tissues decreased month by month [60,110]. However, in mid-summer, the density of active RCs in damaged trees was significantly lower while at the end of autumn the opposite trend was observed, i.e. RC/CS_0_ was higher in the pruned trees. This suggests that in early August, the leaf tissue development in PT was affected by stress and perhaps the leaf vitality was prolonged until late October.

In the face of rapid RC/CS_0_ decrease in October, the shifted values of ABS/RC, TR_0_/RC and DI_0_/RC indicate the imbalance between RCs’ activity and PSII antenna capacity. This imbalance was more pronounced in control trees in which the leaf senescence was more advanced [110]. On the other hand, ET_0_/RC diminished gradually towards the end of the growing season [110]. This trend was more pronounced in the control trees suggesting earlier senescence processes there compared to pruned trees [110]. Previous research by Yordanov et al. [111] showed that removal of younger, i.e. newly emerged leaves leads to senescence delay in primary, i.e. older leaves, and severe defoliation can reverse senescence processes in leaves which typically should have a shortened lifespan. Thus, it can be speculated that the partial canopy loss extended photosynthetic activity in pruned trees in October was due to the delayed leaf senescence. To summarise, chlorophyll fluorescence results confirm that the severe damage in the crown structure evoked the intensified development of leaves and the adjustment of photosynthetic apparatus to maintain proper sugar supply for the entire tree organism. This is consistent with carbon allocation models developed based on the sink: source theory [89]. It should be made clear that the multi-parametric approach in the case of ChF analysis allows the identification of the particular phenomena between PSII and PSI contributing to compensatory photosynthesis. Tis study found that the enhanced probability of electron transport within the plastoquinone pool (from Q_A_ to Q_B_) [112] plays an important role. This may be seen particularly when analysing data collected in August. PI_ABS_ was higher in pruned trees although F_v_/F_m_ was lower, thus higher ET_0_/TR_0_ contributed to significantly higher PI_ABS_.

The evaluation of the PT vitality using Roloff’s classification was problematic because of the atypical architecture of the crown, due to sprouts in newly built or rebuilt parts. In this study, heavy pruning probably interrupted the auxin flow from the terminal buds [113,114], this type of situation has been shown to be a significant promoter of epicormic sprouting [80,115]. The control of epicormic branching is attributed to auxin production in dominant meristems that limits sprouting and development of subordinate buds [113–118]. In the examined trees the removal of main branches caused extensive sprouting. Former research found low tree vigour as a major factor that stimulated epicormic bud sprouting [82,88,119–121]. In this study, fully vigorous trees were heavily pruned, which is perceived to cause a reduction of vigour by removing leaf area. Toussaint et al. [122] contrasted the costs of routine pruning to those associated with topping for common lime (*Tilia* × *Europaea*) street trees in France and found topping both more expensive and more damaging in the long term. Even though tree species show differences in the susceptibility to cavity formation [43] and *Tilia sp*. is perceived as relatively resistant, wounds and necrosis on the trunk caused by cuts were already the locations of observed parasitic fungi, which increased the risk of trunk breakage or tree fall in the root system. PT require an energy investment in CODIT barriers formation and the long-term perspective, this may lead to an increased risk for people and property.

## Conclusions

Pruning treatment which suppresses the primary axis of trees without providing a substitute is harmful in many ways. Severe topping induces changes in a tree growth pattern by increasing the release of sprouting. It also evokes intensified leaf development and photosynthetic apparatus adjustment to assure a higher sugar supply for the damaged organism. Analysis of chlorophyll fluorescence has shown that the necessity for regeneration of the lost canopy triggers the photochemical processes in the light-dependent phase of photosynthesis. The increased linear electron transport is one of the ways of photosynthetic adjustment to higher sugar demands. Despite these efforts towards crown regeneration, improper heavy pruning may result in wounds and necrosis of the trunk and limbs. Consequently, parasitic fungi may easily infect the trunks causing wood decay. This results in the loss of mechanical strength of trunks and limbs in the area of wounds in the following years and evokes a higher risk of the trunk or large branch failure and dieback in mature trees, which was observed in this study. Crown cuts of more than 50% caused the destruction and asymmetric shape of trees. Consequently, deformation of the alley structure was observed.

## Supporting information

S1 Table Tree canopy shape changes and symptoms of increased risk level caused by pruning. (TIF)

S2 Table Supporting information KrynkaPrunedTilia_ChF_raw_data2016. (XLS)
