# Supplementary figures and images for: Impact of heavy pruning on development and photosynthesis of Tilia cordata Mill. trees
Source: PLoS One. 2021 Aug 23;16(8):e0256465. doi: 10.1371/journal.pone.0256465 (PMC8382193; doi:10.1371/journal.pone.0256465)

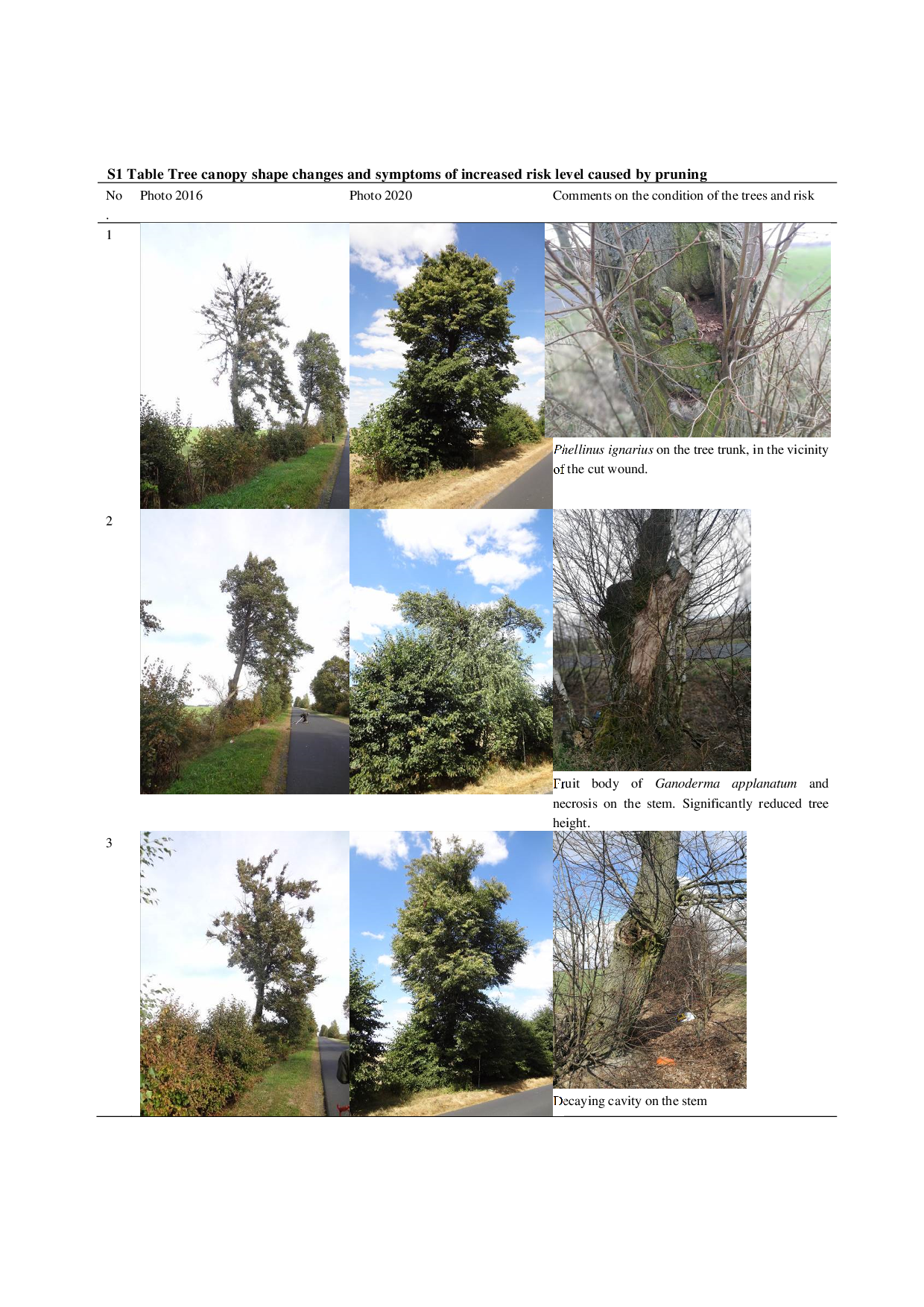

Supplement: S1 Table — (TIF) [file pone.0256465.s001.tif]
